# A Compensatory U1snRNA Partially Rescues FAH Splicing and Protein Expression in a Splicing-Defective Mouse Model of Tyrosinemia Type I

**DOI:** 10.3390/ijms21062136

**Published:** 2020-03-20

**Authors:** Dario Balestra, Daniela Scalet, Mattia Ferrarese, Silvia Lombardi, Nicole Ziliotto, Chrystal C. Croes, Naomi Petersen, Piter Bosma, Federico Riccardi, Franco Pagani, Mirko Pinotti, Stan F. J. van de Graaf

**Affiliations:** 1 Department of Life Sciences and Biotechnology, University of Ferrara, 44121 Ferrara, Italy; scldnl@unife.it (D.S.); frrmtt1@unife.it (M.F.); lmbslv@unife.it (S.L.); zltncl1@unife.it (N.Z.); pnm@unife.it (M.P.); 2 Tytgat Institute for Liver and Intestinal Research, Academic Medical Center, University of Amsterdam, 1105 AZ Amsterdam, The Netherlands; chrystal.croes@outlook.com (C.C.C.); n.petersen@amc.uva.nl (N.P.); p.j.bosma@amc.uva.nl (P.B.); k.f.vandegraaf@amc.uva.nl (S.F.J.v.d.G.); 3 Department of Gastroenterology and Hepatology, Amsterdam Gastroenterology and Metabolism, Academic Medical Center, University of Amsterdam, 1105 AZ Amsterdam, The Netherlands; 4 Human Molecular Genetics, International Centre for Genetic Engineering and Biotechnology, 34149 Trieste, Italy; Federico.Riccardi@icgeb.org (F.R.); pagani@icgeb.org (F.P.); 5 LTTA, University of Ferrara, 44121 Ferrara, Italy

**Keywords:** FAH, fumarylacetoacetate hydrolase deficiency, Tyrosinemia type I, aberrant splicing, mouse models, U1snRNA, RNA therapeutics

## Abstract

The elucidation of aberrant splicing mechanisms, frequently associated with disease has led to the development of RNA therapeutics based on the U1snRNA, which is involved in 5′ splice site (5′ss) recognition. Studies in cellular models have demonstrated that engineered U1snRNAs can rescue different splicing mutation types. However, the assessment of their correction potential in vivo is limited by the scarcity of animal models with the targetable splicing defects. Here, we challenged the U1snRNA in the *FAH5961SB* mouse model of hepatic fumarylacetoacetate hydrolase (FAH) deficiency (Hereditary Tyrosinemia type I, HT1) due to the *FAH* c.706G>A splicing mutation. Through minigene expression studies we selected a compensatory U1snRNA (U1^F^) that was able to rescue this mutation. Intriguingly, adeno-associated virus-mediated delivery of U1^F^ (AAV8-U1^F^), but not of U1^wt^, partially rescued FAH splicing in mouse hepatocytes. Consistently, FAH protein was detectable only in the liver of AAV8-U1^F^ treated mice, which displayed a slightly prolonged survival. Moreover, RNA sequencing revealed the negligible impact of the U1^F^ on the splicing profile and overall gene expression, thus pointing toward gene specificity. These data provide early in vivo proof-of-principle of the correction potential of compensatory U1snRNAs in HTI and encourage further optimization on a therapeutic perspective, and translation to other splicing-defective forms of metabolic diseases.

## 1. Introduction

Mutations leading to aberrant pre-mRNA splicing account for a significant proportion of human genetic disorders [1] and includes changes at the 5′ or 3′ splice sites (ss) but also within exons. The splicing process is a very complex and finely regulated mechanism with several sequence elements and regulatory factors involved [2]. The extensive studies in the field and the elucidation of the mechanisms underlying pathological splicing have led to strategies to modulate it for therapeutic purposes. Among them, the use of the small nuclear ribonucleoprotein U1 (U1snRNP) that, in the earliest splicing step, plays a key role in the exon definition by mediating the recognition of the 5′ss through base pair complementarity with its RNA component (U1snRNA) [3]. Variants of the U1snRNA with increased complementarity with the 5′ss of the defective exon (compensatory U1snRNA), or targeting the downstream intronic sequences (Exon specific U1snRNA, ExSpeU1), have shown the ability to rescue mRNA splicing in the presence of disease-causing mutations at 5′ss, 3′ss or within exons [4,5,6,7,8,9,10,11,12,13,14,15,16,17,18,19]. While the correction effect has been clearly shown in several cellular models of human disease the evaluation of their therapeutic potential requires investigations in animal models harboring the disease-causing splicing mutations, which are very rare. The expression of the splicing-defective human transgene in wild-type mice has been previously exploited to create surrogate models of coagulation factor VII or IX deficiency [7,12], which demonstrated the correction ability of the U1snRNA variants. Only recently it was shown that the delivery of an engineered U1snRNA by adeno-associated virus (AAV) rescues splicing and protein expression and, most importantly, the disease phenotype and survival in a mouse model of spinal muscular atrophy (SMA) [20].

Here, we challenged the U1snRNA-based correction approach on *FAH5961SB* mice, which were affected by the *FAH* c.706G>A mutation causing complete exon 8 skipping and recapitulated the phenotypic characteristics of patients with acute Hereditary Tyrosinemia type I (HT1) [21].

HT1 is an autosomal recessive disorder (OMIM #276700; incidence 1:100,000) due to mutations in the *FAH* gene encoding the hepatic fumarylacetoacetate hydrolase, the last enzyme of the tyrosine degradation pathway. FAH deficiency in mice and humans is associated with progressive liver disease, or severe liver failure in the acute form, and a secondary renal tubular dysfunction due to accumulation of tyrosine/tyrosine metabolites. At present, treatment with 2-(2-nitro-4-trifluoromethylbenzoyl)-1,3-cyclohexanedione (Nitisinone or NTBC), often combined with a low-protein diet, allows patients and also *FAH5961SB* mice to survive [22].

By exploiting the HT1 mouse model, we demonstrated that the AAV-mediated delivery of a compensatory U1snRNA variant to the liver partially rescued FAH expression at RNA and protein levels, thus providing an early in vivo proof-of-principle of correction efficacy of tailored RNA-based strategies for metabolic disorders caused by aberrant splicing.

## 2. Results and Discussion

### 2.1. Identification of Active U1snRNA Variants by Minigene Assays

**Figure 1:**
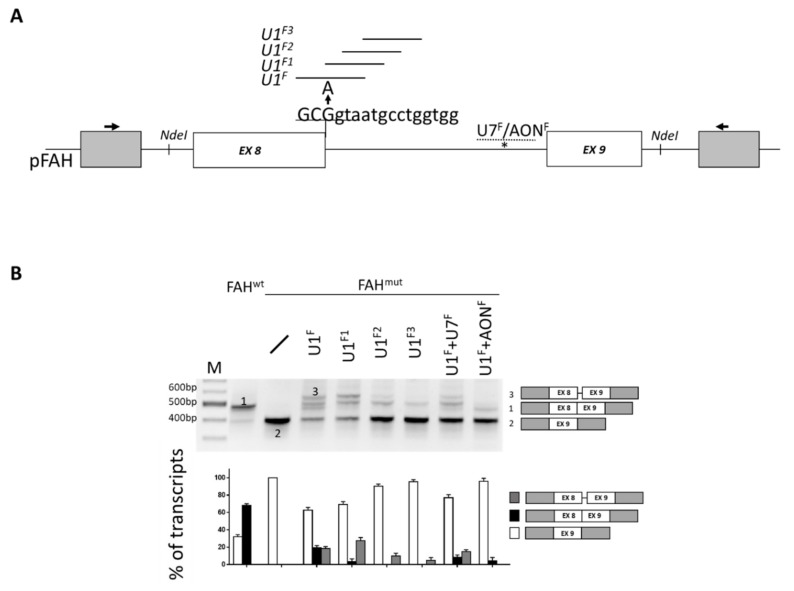
The mouse *FAH* c.706G>A mutation can be rescued by engineered U1snRNAs. (**A**) Schematic representation of the mouse *FAH* minigene with exonic and intronic sequences represented by boxes and lines, respectively (not in scale). Exonic and intronic nucleotides are indicated in upper and lower cases respectively. The nucleotide change (G>A) leading to the FAH deficiency in *FAH5961SB* mice is indicated (arrow). The targeting regions of the engineered U1snRNAs or the U7snRNA/AON^F^ are reported as continuous and dotted lines, respectively. The cryptic 5′ss at position +63 in intron 8 is indicated by an asterisk. (**B**) *FAH* splicing patterns in Hepa1-6 cells transiently transfected with the wild-type (FAH^wt^) or mutated (FAH^mut^) minigenes, alone or in combination with U1/U7snRNA or the AON^F^. The schematic representation of the transcripts (numbers 1 to 3, with exons not in scale) is reported on the right. Amplified products were separated on 2% agarose gel. M, 100 bp molecular weight marker. The lower panel reports the evaluation of amplicons by denaturing capillary electrophoresis (see Figure S1A). Histograms report the relative percentage of each transcript expressed as mean ± standard deviation (SD).

To evaluate *FAH* mRNA splicing modulation, we expressed the FAH minigene spanning intron 7 through 9 (Figure 1A) into Hepa1-6 cells. As shown in Figure 1B, the wild-type minigene (FAH^wt^) processing mainly resulted in correctly spliced transcripts (68 ± 2%) but was also associated with appreciable levels of aberrant transcripts arising from exon 8 skipping (32 ± 2%). This observation supports a poor definition of exon 8. As expected, the c.706G>A mutation led to a virtually complete exon 8 skipping, in accordance with the causative nature of this nucleotide change. In an attempt to force exon 8 inclusion, we designed U1sRNA variants targeting the defective *FAH* 5′ss (U1^F^) or downstream intronic sequences (U1^F1^, U1^F2^, U1^F3^). In co-transfection experiments, all U1snRNA variants led to the synthesis of transcripts with a size compatible with correct processing, and also to forms derived from the usage of the intronic cryptic 5′ss at position +63 (Figure 1A, asterisk). To better define the splicing patterns, the amplicons were fluorescently labelled and analyzed by denaturing capillary electrophoresis, which prevents the confounding effect of hetero-duplex molecules. Inspection of electropherograms revealed that only the compensatory U1^F^ and the ExSpe U1^F1^ re-directed the spliceosome to the mutated 5′ss, thus appreciably rescuing *FAH* mRNA processing (Figure S1A). In particular, correct transcripts accounted for 19.1 ± 2.6% and 3.4 ± 3% of all transcripts for U1^F^ and U1^F1^, respectively. Conversely, the U1^F^, designed on the mutated 5′ss, was ineffective in the wild-type context (Figure S1B).

We subsequently tested the hypothesis that the correction efficiency would be increased by counteracting the use of the cryptic intronic 5′ss. For this purpose, we exploited well-established antisense molecules such as a U7snRNA (U7^F^) or an oligonucleotide (AON^F^) to target and mask it (Figure 1A). Notably, the U1^F^ in combination with the U7^F^ or the AON^F^ remarkably decreased the cryptic 5′ss usage (from 18.2 ± 2.5% to 14.8 ± 2.1% or 0%, respectively) (Figure 1B and Figure S1A). However, this combined approach failed to appreciably increase the proportion of correct transcripts (19.1 ± 2.6 to 8.2 ± 2.9% or 4.1 ± 3.9%, respectively).

Overall, these data provided evidence for the U1snRNA-mediated rescue of *FAH* splicing and allowed selection of the most-active U1^F^ for the in vivo challenge.

### 2.2. Effects on FAH Expression of the Administration of the U1^F^ in Splicing-Defective HT1 Mice

**Figure 2:**
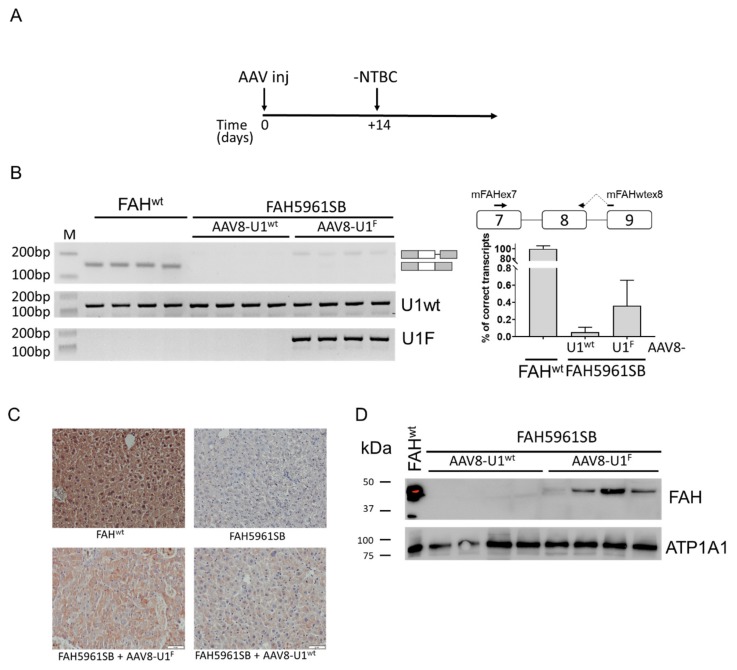
The compensatory U1^F^ partially rescues *FAH* expression *in vivo*. (**A**) Schematic representation of the protocol designed to perform the experiments in mice and exploiting the AAV8-mediated delivery of the U1snRNAs. Mice, kept on NTBC in their drinking water, were injected with 1*10^13^ vg/kg of body weight of AAV8-U1^F^ or AAV8-U1^wt^ and transferred to normal drinking water without NTBC (-NTBC) 14 days later (+14). (**B**) *FAH* splicing pattern profiles in mouse livers, together with the schematic representation of transcripts, reported on the right side. The quantification of correctly spliced *FAH* transcript by qPCR is indicated on the right, with the schematic organization of *FAH* gene region and the exploited primers (on top). Results are reported as percentage of correctly spliced transcripts (mean ± SD). (**C**) Immunohistochemical analysis of FAH expression in liver sections of wild type (FAH^wt^) and *FAH5961SB* mice, either untreated or treated with AAV8-U1^wt^ or AAV8-U1^F^. Representative examples of liver sections stained with a specific anti-FAH antibody (brown). Images are taken at 20× magnification. Scale bar, 50 µm. (**D**) Western blotting analysis through a specific anti-FAH antibody in liver homogenates of wild type (FAH^wt^) and *FAH5961SB* mice treated with AAV8-U1^wt^ or AAV8-U1^F^. The mouse ATPase Na^+^/K^+^ Transporting Subunit Alpha 1 (*ATP1A1*) was exploited as load control. The protein marker, reporting the molecular size of bands, is reported on the left.

Prompted by the in vitro findings, we challenged the U1^F^ in *FAH5961SB* mice, with a focus on *FAH* expression at the RNA and protein level in hepatocytes. As an added value, we also monitored the time for mice to reach the humane endpoint upon removal from drinking water of NTBC, which is commonly used to avoid the severe HT1 phenotype. As shown in Figure 2A, adult *FAH5961SB* mice were initially kept with NTBC, injected with AAV8-U1^wt^ or AAV8-U1^F^ at 1 × 10^13^ viruses/kg and placed on NTBC-free drinking water 14 days post injection (Figure 2A), in conjunction with the expected peak of expression of the AAV8-delivered gene [23]. Mice injected with the control AAV8-U1^wt^ remained at constant weight during the first week and started losing weight soon afterwards. These mice (*n* = 17) reached the humane endpoint within 21 days and 70% within 19 days. Mice treated with AAV8-U1^F^ (*n* = 20) reached the humane endpoint at day 21 (40%) and 25 (15%) (Figure S2A; *p* = 0.0028 Mantel–Cox test and *p* = 0.0310 Gehan–Breslow–Wilcoxon test). At the time of sacrifice, the levels of alanine aminotransferase enzyme (ALAT), a parameter of liver damage, were comparable in the experimental groups. This suggested that the mice died from the same underlying pathology, in line with the described consequences of stopping with NTBC supplementation in *FAH5961SB* mice.

Before evaluating the impact of the U1^F^ delivery on *FAH* mRNA and protein levels in hepatocytes we assessed the transduction efficiency as well as the U1snRNA expression.

The immune-histochemical analysis revealed that the GFP staining, exploited as a marker of viral transduction, was comparable among mice irrespectively of the AAV8-U1 injected and not homogeneously distributed, with large variations in the number of GFP-positive hepatocytes across liver sections (Figure S2B), as previously observed by others [7,24,25]. This finding was consistent with the AAV gene copy number that appeared to be comparable among experimental groups (Figure S2C). On the other hand, the expression of the U1^F^ through appropriately designed primers was clearly detectable only in *FAH5961SB* mice injected with AAV8-U1^F^ (Figure 2B, left panel). In these mice, but not in those treated with the AAV8-U1^wt^, we detected transcripts resulting from the usage of the cryptic 5′ss, a side effect of the U1^F^ also observed with minigenes. The absence of the exon 8 skipped form in both experimental conditions, likely attributable to the occurrence of non-sense mediated decay (NMD), prevented a reliable quantification of all transcripts, which led us to focus on the correctly spliced form only. The exploitation of a sensitive qPCR confirmed negligible amounts, if any, of correct transcripts in control mice treated with AAV8-U1^wt^. Noticeably, treatment with AAV8-U1^F^ resulted in low but detectable levels of correct transcripts, which ranged from 0.3% to 0.8% of those measured in wild-type mice (Figure 2B, right panel). Consistently, the immunostaining in liver sections from *FAH5961SB* mice injected with AAV8-U1^wt^ did not reveal an appreciable FAH protein expression. In contrast, FAH protein was detected at low to intermediate intensity in approximately half of the hepatocytes of AAV8-U1^F^ treated mice (Figure 2C). These findings were corroborated by Western blotting analysis in liver homogenates through which the FAH protein was detected at ~47 kDa in the wild-type mice and not in *FAH5961SB* mice. Injection of AAV8-U1^F^, but not of AAV8-U1^wt^, led to appreciable *FAH* expression in a subset of the treated mice (Figure 2D and Figure S2D). It is worth noting that only a fraction of hepatocytes has been transduced by AAV8 and therefore the comparison of the *FAH* expression in the *FAH5961SB* mice treated with AAV8-U1^F^ with those in normal mouse liver leads to a remarkable underestimation of the correction effect.

Altogether these data indicate that the AAV8-mediated delivery of the compensatory U1^F^ results in low but appreciable rescue of *FAH* splicing that in turn leads to the appearance of the FAH protein in hepatocytes. We were aware that in the rescued transcripts the mutation in the last exonic nucleotide introduced the Ala236Thr substitution. However, the amino acid change did not seem to remarkably impair protein synthesis or induce intracellular degradation, as supported by the detection of FAH protein upon AAV8-U1^F^ treatment. On the other hand, the Ala236Thr change could impair FAH activity, an effect hardly assessable in our in vivo model because of the very low expression levels, which would have required the biochemical characterization of the FAH variant. However, although further in vivo investigations are required, it is tentative to speculate that the rescued FAH protein isoform retained, at least to some extent, enzymatic activity that could contribute to explaining the slight prolongation of the survival curve in AAV8-U^F^ treated mice.

### 2.3. Specificity of the Compensatory U1^F^

As engineered U1snRNAs could theoretically affect the splicing machinery in general, off-target effects need to be carefully addressed. Therefore, we evaluated changes in the alternative splicing profile and in the global gene expression in liver specimens from *FAH5961SB* mice treated with AAV8-U1^F^ or, as control, AAV8-U1^wt^.

**Figure 3:**
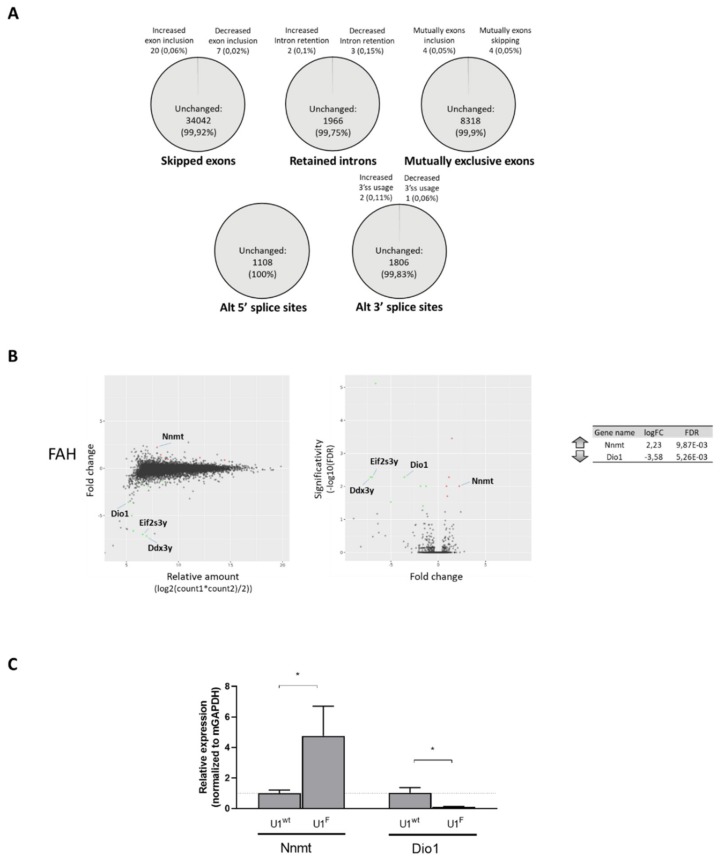
The compensatory U1^F^ does not have a widespread effect on splicing and overall gene expression. (**A**) Fraction of each category (SE, RI, MXE, A5SS and A3SS) of Gencode-annotated splicing events showing increased or decreased alternative isoform use in hepatocytes from *FAH5961SB* mice treated with AAV8-U1^F^ as compared with those treated with AAV8-U1^wt^. For each category, the number and the percentage (%) of events is indicated in the graph (FDR ≤0.05; Inclusion Level Difference ≤−0.2 or ≥0.2). (**B**) Comparison of the gene expression profile in hepatocytes from *FAH5961SB* mice injected with AAV8-U1^F^ as compared with those injected with the control AAV8-U1^wt^. Only genes coding for proteins and having a fold change higher than two are shown. MA plot showing the relationship between the average expression value (on the *X*-axis) and the fold change (*Y*-axis) for each gene in the genome (left panel). Volcano plot showing the relationship between the fold-change (on the *X*-axis) and the significance of the differential expression test (*Y*-axis) for each gene (middle panel). Black dots represent the genes that are not significantly differentially expressed, while red and green dots represent the genes significantly UP- and DOWN-regulated, respectively. List of coding genes UP and Down regulated and mapping in autosomes (right panel). Only genes having a fold-change (FC) >2 were reported. Adjusted *p*-values for multiple tests (by the Benjamini–Hochberg procedure) are reported as “false discovery rate” (FDR) value. (**C**) Validation of RNAseq data by qPCR. Histograms report the relative gene expression ± standard deviation (SD) from three independent experiments, normalized for the house keeping mouse *GAPDH*.

Among all identified alternative splicing events, the delivery of AAV8-U1^F^ was associated with a negligible effect (<0.1%) on splicing (Figure 3A), thus supporting the specificity of the compensatory U1^F^ for the mutated 5′ss. In accordance with qPCR analysis, the RNAseq further highlighted the splicing rescue of *FAH* exon 8 upon treatment with the compensatory U1^F^.

Concerning the impact on global gene expression, only a small proportion of the 13000 investigated genes displayed an altered profile. In particular, the delivery of AAV8-U1^F^ resulted in the over- and under- expression of five and eight genes, respectively. However, by considering only protein-coding genes and a fold change higher than two, we observed one over- and three under- expressed genes (Figure 3B). Among them, we selected those displaying the highest expression differences, mapping in autosomes, to avoid Y chromosome-related confounding effects, and containing introns (Figure 3B, right panel) and validated the RNA-seq data by qPCR analysis, which revealed that both had a statistically significant altered expression (Figure 3C).

Taken together our data provide experimental evidence that the compensatory U1snRNA U1^F^ has a very limited off-target effect, which is also consistent with the presence of several endogenous U1snRNA variants that are known to be functional and possess physiological roles [26,27,28].

## 3. Materials and Methods

### 3.1. Creation of Minigene Vectors

To create the pFAH vectors, the genomic region of mouse *FAH* gene was amplified with the high-fidelity PfuI DNA-polymerase (Transgenomic, Glasgow, UK) from genomic DNA of a *CL57BL6* mouse, and cloned into the expression vector pTB [18] through the *NdeI* restriction sites inserted within primers. The genomic region spanning intron 7 through 9 of the mouse *FAH* gene, including 134 and 170 nucleotides of the upstream and downstream introns, was amplified with primer 7F-9R. The *FAH* c.706G>A mutation was inserted by mutagenesis (QuickChange II Site-Directed Mutagenesis Kit, Stratagene, La Jolla, CA, USA). Expression vectors for the U1snRNA and U7snRNA variants were created as previously reported [17]. Sequences of all oligonucleotides are provided in Table S1. All plasmids were validated by direct sequencing.

### 3.2. Expression in Mammalian Cells and mRNA Studies

Mouse hepatoma Hepa1-6 cells were seeded on 12-well plates and transfected with Lipofectamine 2000 reagent (Life Technologies, Carlsbad, CA, USA) as previously reported [29]. Five hundred nanograms of each minigene construct were transfected alone or in combination with a molar excess (1.5x) of the pU1/pU7 plasmids. Total RNA was isolated twenty-four hours post-transfection with Trizol (Life Technologies, Carlsbad, CA, USA), reverse-transcribed with random primers with RT-MLV (Life Technologies, Carlsbad, CA, USA) and amplified using the plasmid-specific primers Alfa and Bra (Table S1). All transcripts were validated by direct sequencing.

### 3.3. Preparation of the AAV Vectors

AAV vectors were produced using a previously described adenovirus-free transient transfection method [30]. In brief, human embryonic kidney cells (HEK293T) were transfected with AAV2 Rep and AAV8 Cap (pDP8.ape, Plasmid Factory, Bielefeld, Germany) and the ITR-flanked transgene expression cassette. After 72 h from transfection, cells were harvested, lysed by two freeze-thaw cycles, and treated with DNAse, RNAse (Roche, Basel, Switzerland) and benzonase (Merck, Darmstadt, Germany). Vectors were then purified by iodixanol gradient ultra-centrifugation. Titers of AAV vector stocks were determined using quantitative real-time polymerase chain reaction (qPCR) with primers directed to the eGFP sequence (Table S1).

### 3.4. Procedures in Mice

The *FAH5961SB* mouse model was obtained from Jackson laboratories (JAX stock #018129, Jackson laboratories, Bar Harbor, ME, USA) and subsequently bred in the Academic Medical Center Amsterdam. The *FAH5961SB* mouse strain has been generated through N-ethyl-N-nitrosourea (ENU) mutagenesis, which led to insertion in the mouse genome of the FAH c.706G>A mutation that affects *FAH* expression. These mice, mimicking acute Tyrosynemia type I, only survive if they are maintained on NTBC-containing drinking water [21]. Wild-type littermates were used as a control. Animals were kept on a 12 h light:12 h dark continuous cycle with ad libitum access to food and water. *FAH5961SB* mice were fed a standard rodent chow and water supplemented with NTBC. Female and male mice were 8–12 weeks old at the start of the experiment when AAV was injected via the tail vein.

For all experiments a body weight loss of 20% of their maximally obtained weight was considered the humane endpoint and mice were sacrificed. At this stage, a blood sample was taken and organs were harvested to be frozen in liquid nitrogen or formalin fixed. The study design and animal care and handling were approved by the Institutional Animal Care and Use Committee of the University of Amsterdam.

### 3.5. Splicing Pattern Analysis in Mouse Hepatocytes

Total RNA, extracted from mouse livers with Trizol (Life Technologies, Carlsbad, CA, USA), was reverse-transcribed with random primers with RT-MLV (Thermo Fisher Scientific, Waltham, MA, USA). Mouse *FAH* splicing pattern analysis was conducted by RT-PCR with primers mFAHex7-mFAHex9 (Table S1), followed by conventional agarose gel electrophoresis. The evaluation of correctly spliced transcripts was also performed on diluted 1:10 cDNA by quantitative PCR (qPCR) with SsoAdvanced Universal SYBER Green Supermix (Bio-Rad, Hercules, CA, USA) according to the supplier’s protocol on a CFX connect qPCR system (Bio-Rad, Hercules, CA, USA) with primers mFAHex7-mFAHwtex8. Each sample was run in duplicate. Cq and melting curves were acquired by use of Bio-Rad CFX Manager 3.1 software (Bio-Rad, Hercules, CA, USA). The mRNA levels were expressed as the relative expression index of 2-ΔΔCt. Values were expressed as mean fold change ± standard error of the mean.

### 3.6. Evaluation of Viral Transduction by Gene Copy Number

Frozen liver tissue was homogenized and total genomic DNA isolated with the use of the Wizard Genomic DNA Purification Kit (Promega, Madison, WI, USA). AAV copy number was evaluated by qPCR as described above. A GFP-specific sequence was amplified with primers GFPF-GFPR (Table S1). AAV8-U1 copy number was calculated on a standard curve created by spiking known amounts of linearized AAV8-U1^wt^ plasmid into 500 nanograms of liver genomic DNA isolated from a saline-injected mouse (lowest limit, 18 AAV8 copies). In particular, one microliter of total DNA sample was used in the absolute qPCR reaction. Each sample was run in duplicate. Samples with an amplification signal below the lowest point of the standard curve were discarded. AAV8 vector copy number was then normalized by the number of double-stranded DNA of diploid genomic equivalent.

### 3.7. Evaluation of FAH Protein Expression

Livers were excised, and segments either fixed in 4% PFA at 4 °C o/n and stored in 70% ethanol at 4 °C until paraffin embedding or snap frozen in liquid nitrogen. Sections from *FAH5961SB* or wild type mice were deparaffinized and rehydrated, treated with 0.3% H_2_O_2_ to block endogenous peroxidase and citrate as antigen retrieval and incubated with rabbit anti-FAH (1:200 Origene #TA354752, Origene Global, Rockville, MD, USA) and Poly-HRP-goat secondary antibody (Immunologic DPVB110 HRP) Sections were stained using Vector NovaRed (HRP)substrate kit (#SK-4800, Vector Laboratories, Burlingame, CA, USA), counterstained with haematoxylin and mounted with Vectamount (#ZD0104, Vector Laboratories, Burlingame, CA, USA)). One hundred microgram protein was loaded per lane and analyzed by Western blotting using the above reported antibodies (anti-FAH at 1:1000 dilution).

### 3.8. RNA Sequencing Data Generation

The liver RNA from *FAH5961SB* mice treated with AAV-U1^F^ or AAV-U1^wt^, as the control, was purified with the TRIreagent (Invitrogen, Carlsbad, CA, USA). The quality of total RNA was assessed using Agilent RNA 2100 Eukaryote Total RNA Nano Bioanalyzer microfluidic chips (Agilent Technologies) and a Qubit 2.0 dsDNA HS assay (Life Technologies, Carlsbad, CA, USA). The template DNA molecules suitable for cluster generation were prepared from 500 ng of total RNA samples using the NEBNext^®^ Poly(A) mRNA Magnetic Isolation Module followed by NEBNext^®^ Ultra™ II RNA Library Prep Kit (Illumina Inc., San Diego, CA, USA) for Illumina according to the manufacturer’s instructions. The size distribution of the libraries was estimated by electrophoresis on Tapestation High Sensitivity D1000 Assay (Agilent Technologies, CA, USA). Libraries were quantified using the KAPA Library Quantification Kit (KK4824, Kapa Biosystems, Boston, MA, USA). The libraries were pooled at equimolar concentrations and diluted before loading onto the flow cell of the HiSeq 2x150 (Illumina Inc., San Diego, CA, USA) for both clustering and sequencing. The libraries were extended and bridge-amplified to create a single sequence. Amplified clusters in the flow cell were then sequenced with 150-bp paired-end reads using the TruSeq Rapid SBS Kit––HS (Illumina Inc., San Diego, CA, USA). Real-time image analysis and base calling were performed on a HiSeq 2500 instrument (Illumina Inc., San Diego, CA, USA) using the recommended sequencing control software. Bcl2fastq software (v2.19.1.403) (Illumina Inc., San Diego, CA, USA) was used for de-multiplexing and production of FASTQ sequence files. FASTQ raw sequence files were subsequently quality checked using FASTQC software version 0.11.3 (http://www.bioinformatics.bbsrc.ac.uk/projects/fastqc) (Babraham Bioinformatics, Babraham, UK).

### 3.9. RNAseq Data Analysis and Validation

The resulting set of trimmed reads were then mapped onto GRCm38/mm10 *Mus Musculus* reference genome (http://genome.ucsc.edu/) using the Spliced Transcripts Alignment to a Reference (STAR) algorithm (version 2.5.2b) [31]. Differential gene expression analysis was performed using FeatureCounts (version 1.5.1) (Walter and Eliza Hall Institute, Parkville, VIC, Australia).

Normalization was applied to the raw fragment counts by using the Trimmed Mean of M-values (TMM) normalization and Fragments Per Kilobase Million (FPKM) normalization. All the statistical analyses were performed with R with the packages HTSFilter and edgeR. The following step involves the removal of the not expressed genes and the ones showing too much variability. The HTSFilter package was chosen for this scope, which implements a filtering procedure for replicated transcriptome sequencing data based on a Jaccard similarity index. The Trimmed Means of M-values (TMM) normalization strategy was used. The overall quality of the experiment was then evaluated, based on the similarity between replicates, by a Principal Component Analysis (PCA) using the normalized gene expression values as input. An MA plot and a Volcano plot were also generated for each comparison. The estimated *p*-values for each gene were adjusted using the Benjamini–Hochberg method (FDR). Features with an FDR value ≤0.05 were considered as having a significant altered expression. A panel of selected differentially expressed genes were validated by qPCR as mentioned above (see primers in Table S1).

For genome-wide splicing analysis, BAM files produced from STAR mapping were input into rMATS [32], using GRCm38/mm10 *Mus musculus* as the reference genome. For detection of alternative splicing (AS) patterns, mouse annotations were generated containing all consecutive spliced and unspliced exon-intron-exon triads from mm10 (version M23). Five basic types of AS were analyzed: skipped exons (SE), retained introns (RI), mutually exclusive exons (MXE), alternative 5′ splice sites (A5SS) and alternative 3′ splice sites (A3SS). Read coverage was based on actual reads as used in [33]: SE, RI and MXE types with an actual reads mapping to all exclusion splice junction ≥ 20 were considered, whereas for A5SS and A3SS types, ≥ 40 actual reads mapping to the sum of all splice junctions involved in the specific event were considered. Estimated FDR values for each gene were adjusted using the Benjamini–Hochberg method. The threshold parameters were set at FDR value ≤ 0.05 and absolute Inclusion Level Difference (ΔΨ) ≤ −0.2 or ≥ 0.2.

## 4. Conclusions

In this study, through the exploitation of a pre-existing mouse model of acute HT1 directly linked to a splicing defect, we provided the early proof-of-principle that a compensatory U1snRNA can rescue *FAH* splicing and protein expression in vivo. Moreover, the compensatory U1^F^, albeit targeting the 5′ss, appeared to guarantee a remarkable gene specificity, comparable to that previously demonstrated for the Exon Specific U1snRNA (ExSpeU1) targeting poorly conserved intronic sequences [13,20]. This observation strengthens the potential of the compensatory U1snRNA variants that, in most exon contexts, are much more effective than ExSpeU1s.

These data provide early in vivo proof-of-principle of the correction potential of compensatory U1snRNAs in HTI and encourage further optimization on a therapeutic perspective, and translation to other splicing-defective forms of metabolic diseases.

## Supplementary Materials

Supplementary materials can be found at https://www.mdpi.com/1422-0067/21/6/2136/s1.
