# Supplementary material for: A Compensatory U1snRNA Partially Rescues FAH Splicing and Protein Expression in a Splicing-Defective Mouse Model of Tyrosinemia Type I
Source: Int J Mol Sci. 2020 Mar 20;21(6):2136. doi: 10.3390/ijms21062136 (PMC7139742; doi:10.3390/ijms21062136)
Supplement: Supplementary file 1 [file ijms-21-02136-s001.pdf]

Figure S1

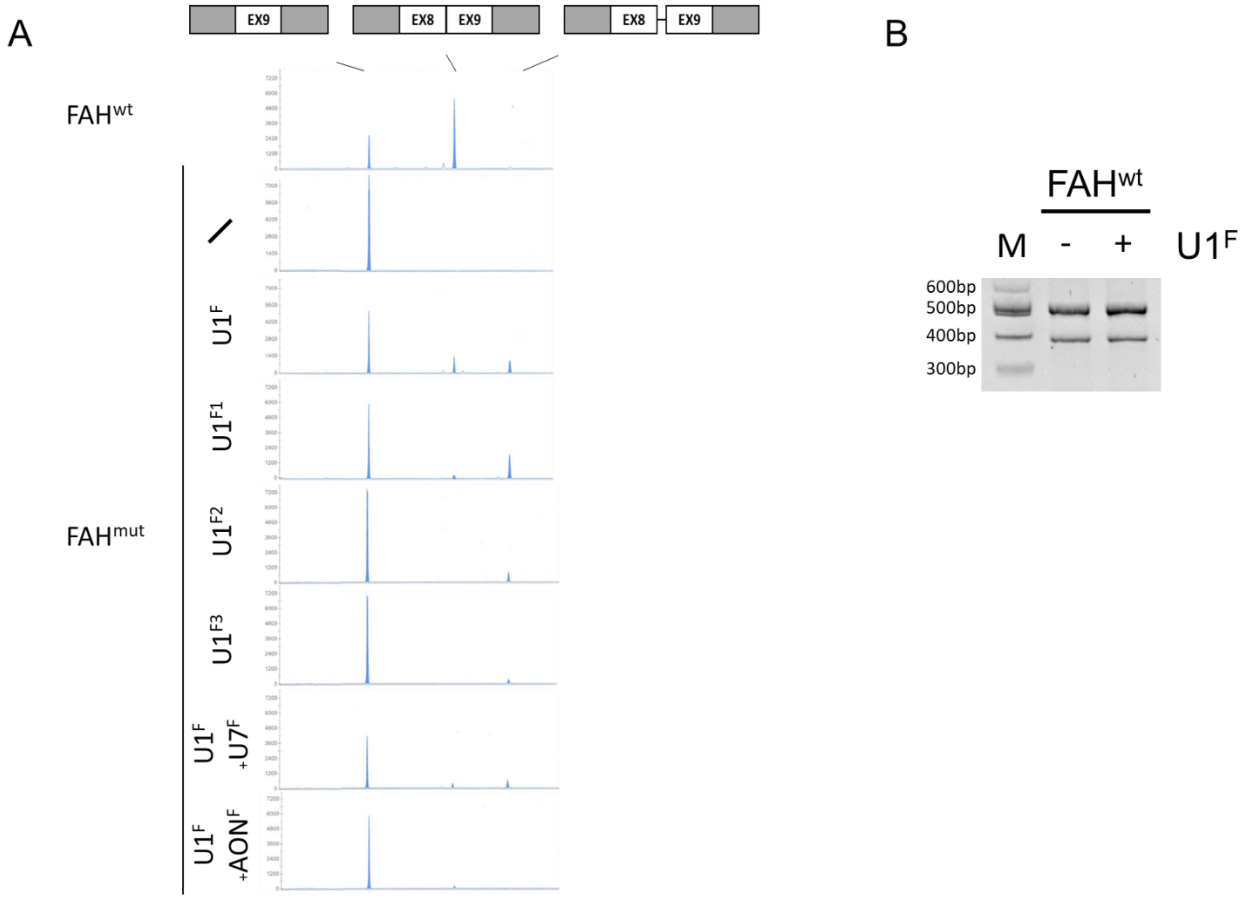

Figure S1. (A) Evaluation of *FAH* minigene splicing patterns by denaturing capillary electrophoresis of fluorescently-labelled PCR products. The amount of transcripts is represented by the area under each peak, and the analysis is shown in figure 1B. The scheme of transcripts is reported on top. RFU: Relative Fluorescence Units **(B)** FAH splicing patterns in Hepa1-6 cells transiently transfected with the wild-type (FAH^{wt}) minigene, alone or in combination with $U1^F$. Amplified products were separated on 2% agarose gel. M, 100 bp molecular weight marker.

Figure S2

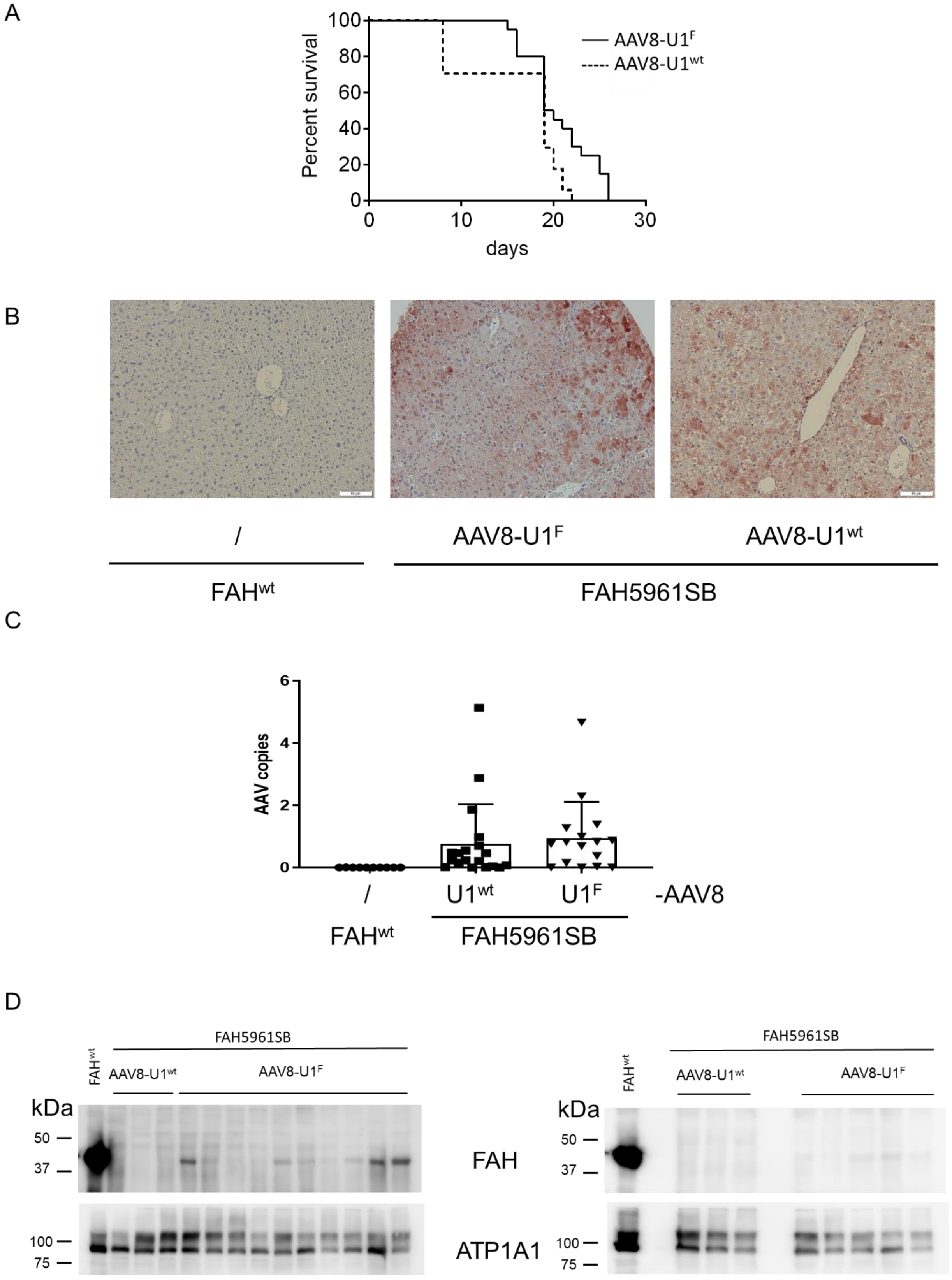

Figure S2. (A) Survival curve of *FAH5961SB* mice injected with the AAV8-U1wt (dotted line) or AAV8-U1F (continuous line) and kept on normal drinking water (-NTBC). The time until the mice reached the humane

endpoint is shown. **(B)** Examples of liver sections from *FAH5961SB* mice injected with the AAV8-U1^{wt} or AAV8-U1^{F}, upon staining with an anti-GFP antibody (brown). Images are taken at 20× magnification. Scale bar, 50 μm. **(C)** Evaluation of liver transduction efficiency by evaluation of AAV8 gene copy number. **(D)** Western blotting analysis through a specific anti-FAH antibody in liver homogenates of wild type (FAH^{wt}) and *FAH5961SB* mice treated with AAV8-U1^{wt} or AAV8-U1^{F}. The mouse ATPase Na^+/K^+ Transporting Subunit Alpha 1 (*ATP1A1*) has been exploited as load control. The protein marker, reporting the molecular size of bands, is reported on the left.

Table S1

Oligonucleotide		Sequence (5′→3′)
Creation of mouse *FAH* minigene		
**7F**	forward	tgaccatatgagggaagtaatgccaggt
**9R**	reverse	tacgcatatgcttatagcctctattccaa
Mutant mouse *FAH* minigene		
**FAH mut F**	forward	gaacgactggagcagtaatgcctggtg
**FAH mut R**	reverse	caccaggcattactgctccagtcgttc
Engineered U1snRNA variants for mouse *FAH* 5′ss		
**$U1^{F}$ F**	forward	aggcccaagatctgatCATTACTGCgcaggggagataccat
**$U1^{F1}$ F**	forward	aggcccaagatctgatAGGCATTACgcaggggagataccat
**$U1^{F2}$ F**	forward	aggcccaagatctgatCCAGGCATTgcaggggagataccat
**$U1^{F3}$ F**	forward	aggcccaagatctgatCCACCAGGCgcaggggagataccat
Engineered U7snRNA variant for mouse *FAH* 5′ss		
**U7F**	forward	acagaggcctttccgcagtcccatacccaactcaatttttggag
Plasmid-specific primers used to analyze splicing pattern		
**alfa**	forward	caacttcaagctcctaagccactgc
**bra**	reverse	taggatccggtcaccaggaagttggttaaatca
Primers used to assess in vivo splicing		
**mFAHex7**	forward	actcttagacatggagttggaaatg
**mFAHex9**	reverse	gtactcccattgctggatgtct
Primers used to evaluate the correctly spliced transcripts		
**mFAHwtex8**	reverse	ttgctggatgtctcgtgcgc
Primers used to evaluate U1 expression		
**$U1^{wt}$Ex**	forward	agatctcatacttacctg
**$U1^{F}$Ex**	forward	agatctgatcattactgc
**U1Ex**	reverse	gaacgcagtcccccactaccac
Primers used to evaluated RNAseq data		
**Nnmt**	forward	atattctgcctgggtgctgt
	reverse	gtcaaaggctcctggttcct
**Dio1**	forward	gggatttcattcaaggcagca
	reverse	acgttgttcttaaaagcccagc
**GAPDH**	forward	cgaccactttgtcaagctcat
	reverse	ccctgttgctgtagccaaatt
Primers used to evaluate the titer of AAV vectors		
**eGFP**	forward	gcggggcaagtgaccgtgtg
	reverse	tgcgcaaacccagggctgcc
Primers used to evaluate the AAV8 gene copy number		
**GFP**	forward	catggtcctgctggagttc
	reverse	gtcaggtcagcttggtcttc

The NdeI restriction site is underlined; mutated nucleotides are in red; the replaced 9 nucleotides of 5′ tail of U1snRNA variants are indicated in upper case.
